# Pearls and pitfalls in diagnosing prostate cancer using multiparametric MRI (mpMRI)

**DOI:** 10.1186/1470-7330-15-S1-P37

**Published:** 2015-10-02

**Authors:** JJ Joshi, S Lee, P Acher, SH Liyanage

**Affiliations:** 1 Southend University NHS Hospital Trust, UK

## Learning objectives

Using our experience of over 500 patients who have undergone pre-biopsy mpMRI (T2, DWI +/- DCE) and subsequent transperineal saturation prostate biopsy, we aim to:

• Illustrate the commonly overlooked areas in diagnosing prostate cancer with mpMRI.

• Emphasise technical factors that can contribute to suboptimal image interpretation.

• Highlight normal anatomical structures and non-cancerous abnormalities that mimic tumour.

• Demonstrate the use of mpMRI to direct further management.

## Content organisation

mpMRI may be used in the pre-biopsy setting to determine type of biopsy (targeted vs. transperineal vs. transrectal), and predict biopsy outcome to the extent that biopsies may be avoided altogether. Tumour localisation within the prostate gland aids targeted biopsy and influences treatment (e.g. suitability for nerve sparing or radiation dose escalation). Tumours with unusual appearances and those in uncommon sites hinder MRI interpretation, potentially leading to false-negative or –positive findings. These areas of pitfall can be divided into normal anatomical structures in the peripheral or transitional zones or non-cancerous abnormalities that mimic tumours (e.g. granulomatous prostatitis).

It is also important to acknowledge that mpMRI itself has limitations. Technical challenges in relation to DWI may lower tumour sensitivity due to anatomical distortion, inadequate suppression of benign prostate tissue and suboptimal ADC map windowing.

## Conclusion

It is paramount that radiologists are aware of the commonly missed locations of prostate cancer, tumour mimics and the limitations of mpMRI, particularly in the context of a multidisciplinary team setting. This would serve to improve diagnostic accuracy, target areas for biopsy more precisely and correctly influence management.

